# Amidation of glutamate residues in mycobacterial peptidoglycan is essential for cell wall cross-linking

**DOI:** 10.3389/fcimb.2023.1205829

**Published:** 2023-08-24

**Authors:** Moagi T. Shaku, Karl L. Ocius, Alexis J. Apostolos, Marcos M. Pires, Michael S. VanNieuwenhze, Neeraj Dhar, Bavesh D. Kana

**Affiliations:** ^1^ DSI/NRF Centre of Excellence for Biomedical Tuberculosis (TB) Research, Faculty of Health Sciences, University of the Witwatersrand, National Health Laboratory Service, Johannesburg, South Africa; ^2^ Department of Chemistry, University of Virginia, Charlottesville, VA, United States; ^3^ Department of Chemistry, Indiana University Bloomington, Bloomington, IN, United States; ^4^ Global Health Institute, Ecole Polytechnique Fédérale de Lausanne, Lausanne, Switzerland

**Keywords:** MurT-GatD complex, peptidoglycan biosynthesis, peptidoglycan amidation, peptidoglycan cross-linking, peptidoglycan remodelling

## Abstract

**Introduction:**

Mycobacteria assemble a complex cell wall with cross-linked peptidoglycan (PG) which plays an essential role in maintenance of cell wall integrity and tolerance to osmotic pressure. We previously demonstrated that various hydrolytic enzymes are required to remodel PG during essential processes such as cell elongation and septal hydrolysis. Here, we explore the chemistry associated with PG cross-linking, specifically the requirement for amidation of the D-glutamate residue found in PG precursors.

**Methods:**

Synthetic fluorescent probes were used to assess PG remodelling dynamics in live bacteria. Fluorescence microscopy was used to assess protein localization in live bacteria and CRISPR-interference was used to construct targeted gene knockdown strains. Time-lapse microscopy was used to assess bacterial growth. Western blotting was used to assess protein phosphorylation.

**Results and discussion:**

In *Mycobacterium smegmatis*, we confirmed the essentiality for D-glutamate amidation in PG biosynthesis by labelling cells with synthetic fluorescent PG probes carrying amidation modifications. We also used CRISPRi targeted knockdown of genes encoding the MurT-GatD complex, previously implicated in D-glutamate amidation, and demonstrated that these genes are essential for mycobacterial growth. We show that MurT-rseGFP co-localizes with mRFP-GatD at the cell poles and septum, which are the sites of cell wall synthesis in mycobacteria. Furthermore, time-lapse microscopic analysis of MurT-rseGFP localization, in fluorescent D-amino acid (FDAA)-labelled mycobacterial cells during growth, demonstrated co-localization with maturing PG, suggestive of a role for PG amidation during PG remodelling and repair. Depletion of MurT and GatD caused reduced PG cross-linking and increased sensitivity to lysozyme and β-lactam antibiotics. Cell growth inhibition was found to be the result of a shutdown of PG biosynthesis mediated by the serine/threonine protein kinase B (PknB) which senses uncross-linked PG. Collectively, these data demonstrate the essentiality of D-glutamate amidation in mycobacterial PG precursors and highlight the MurT-GatD complex as a novel drug target.

## 1 Introduction

**Figure 1:**
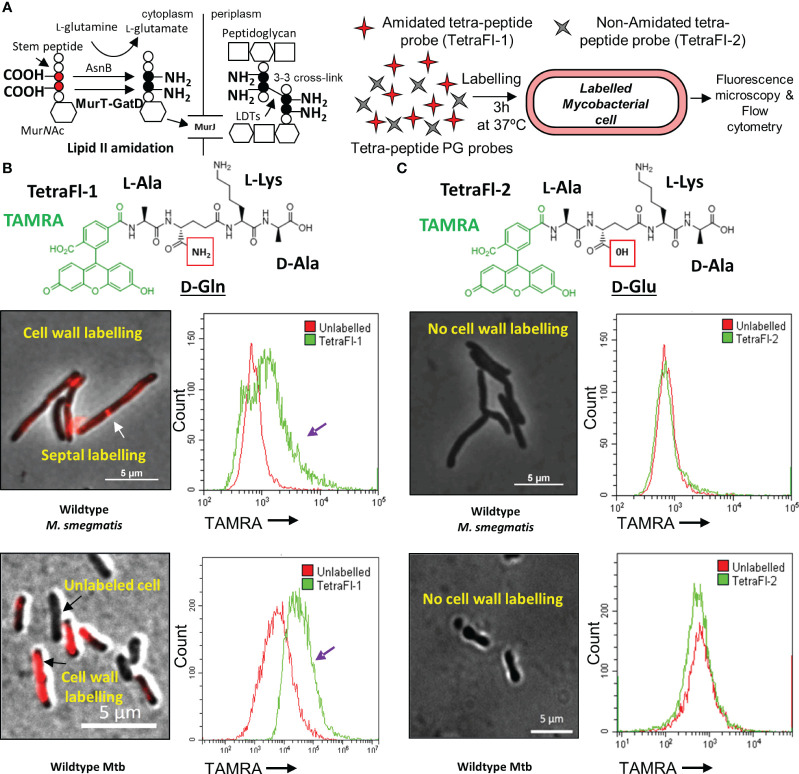
Amidated peptidoglycan stem peptide mimics are incorporated into the mycobacterial cell wall. **(A)** Schematic representation of MurT-GatD and AsnB mediated PG precursor amidation and PG cross-linking. **(B)** Labeling of *M. smegmatis* mc^2^155 and Mtb H37Rv cells with Tetramethylrhodamine (TAMRA)-3-amino-L-alanine-D-glutamine-L-lysine-D-alanine (TetraFl-1). Red box shows the amide group of D-glutamine (D-Gln) in the TetraFl-1 chemical structure. TetraFl-1 labelled *M. smegmatis* mc^2^155 and Mtb H37Rv cells. The change in TAMRA fluorescence intensity during TetraFl-1 incorporation into PG is indicated by the purple arrow in the flow cytometry histograms. White arrow shows septal labelling with TetraFl-1. **(C)** Labeling of *M. smegmatis* mc^2^155 and Mtb H37Rv cells with TAMRA-3-amino-L-alanine-D-glutamate-L-lysine-D-alanine (TetraFl-2). Red box shows the hydroxyl group of D-glutamate (D-Glu) in the TetraFl-2 chemical structure. TetraFl-2 did not label *M. smegmatis* mc^2^155 and Mtb H37Rv cells, indicating that the amidation at D-Gln is required for probe incorporation.

Peptidoglycan (PG) is a heteropolymer composed of glycan strands made up of alternating units of *N*-acetylglucosamine (Glc*N*Ac) linked to *N*-acetylmuramic acid (Mur*N*Ac) cross-linked by stem peptides attached to the lactyl moiety of Mur*N*Ac (Vollmer and Bertsche, 2008). PG biosynthesis is initiated in the bacterial cytoplasm, where a variety of enzymes use carbohydrate monomers and amino acids to build the PG precursor, also known as lipid II (Vollmer and Bertsche, 2008). Lipid II is then modified by enzymes such as NamH, a UDP-*N*-acetylmuramic acid hydroxylase, that catalyzes hydroxylation of the UDP-Mur*N*Ac monomer, resulting in the formation of UDP-*N*-glycolylmuramic acid (UDP-Mur*N*Glyc) (Abrahams and Besra, 2018). The pentapeptide unit of lipid II is also modified at the α-carboxyl group of D-glutamate (D-Glu) by the MurT-GatD complex (Münch et al., 2012; Maitra et al., 2019) and at the α-carboxyl group of *meso*-diaminopimelic by AsnB (Ngadjeua et al., 2018; Pidgeon et al., 2019) (**Figure 1A**). Finally, modified lipid II is translocated to the periplasm by the cell membrane-embedded flippase MurJ and incorporated into the existing PG through transglycosylation and transpeptidation reactions, facilitated by penicillin binding proteins (PBPs) (Vollmer, 2007) (**Figure 1A**). The transpeptidation reactions form either 4-3 cross-links between adjacent PG stem peptides (D-alanine-*meso*-DAP, formed by D,D-transpeptidases) or 3-3 cross-links (*meso*-DAP-meso-DAP, by L,D-transpeptidases [LDTs]). In mycobacteria, 3-3 cross-links constitute the majority of PG cross-links and are required for the maintenance of cell wall integrity during aging of the cell wall (Lavollay et al., 2008; Baranowski et al., 2018; Francis et al., 2023). The Mur*N*Ac/Mur*N*Glyc residues of lipid II are further modified by the addition of an α-L-rhamnopyranose-(1-3)-α-D-NAG-(1-P) unit that links PG to arabinogalactan (Abrahams and Besra, 2018). Considering the essential role of the PG modifications for cell wall integrity, their biological significance and associated pathways in mycobacteria still require elucidation.

Recently, it was demonstrated that a lack of lipid II amidation in PG inhibits cross-linking by PG transpeptidases and this was also confirmed by the use of synthetic PG fragments with amidated D-Glu amidated (i.e. D-Gln) residues (Pidgeon et al., 2019). The lack of PG cross-linking because of reduced PG precursor amidation sensitizes bacteria to β-lactam antibiotics, lysozyme and immunological detection during infection (Wolfert et al., 2007; Figueiredo et al., 2012; Levefaudes et al., 2015; Vijayrajratnam et al., 2016). Ldt_FM_ and Ldt_MT2_ of *Enterococcus faecium* and *Mycobacterium tuberculosis* (Mtb), respectively, are not able to bind either stem peptide derivatives with non-amidated D-Glu as substrates for PG biosynthesis (Ngadjeua et al., 2018). In addition, depletion of AsnB, a glutamate amidotransferase required for *meso*-DAP amidation in Mtb was shown to result in cell death due to defective PG cross-linking (Ngadjeua et al., 2018). Furthermore, the D-Glu amidation on PG peptide stems is required by not only LDTs, but also PBPs for efficient D,D-transpeptidation reactions resulting in the formation of 4-3 cross-links (Zapun et al., 2013). Similarly, deletion of LtsA, a *meso*-DAP amidotransferase in Corynebacteria, caused a reduction in PG amidation at the *meso*-DAP residue. Although not affecting PG cross-linking, this caused increased sensitivity to lysozyme (Levefaudes et al., 2015).

Biochemical studies of the mycobacterial MurT-GatD complex have confirmed a role for this protein complex in mycobacterial PG biosynthesis (Maitra et al., 2021). The MurT-GatD complex catalyzes D-Glu amidation using L-glutamine as an amine donor, thereby converting L-glutamine into NH_3_ and glutamate by GatD, followed by the shuttling of NH_3_ from the GatD active site to the amidotransferase active-site of MurT. Finally, through an ATP-dependent reaction, MurT catalyzes lipid II amidation by transferring NH_3_ to D-glutamate found in the second position of the lipid II stem peptide (Morlot et al., 2018; Nöldeke et al., 2018).

In this work, we used the CRISPRi platform (Rock et al., 2017) to deplete MurT and GatD in *Mycobacterium smegmatis* and showed that this caused cell growth inhibition as a result of expected reduced lipid II amidation and we studied the associated pathways resulting in this phenotype. By using fluorescent tetrapeptide probes, we further confirmed that D-Gln is required for probe incorporation into mycobacterial cells. Lastly, we confirmed the essentiality of the MurT-GatD complex for mycobacterial growth and cell wall stability.

## 2 Materials and methods

### 2.1 Bacterial strains and culture conditions

*E. coli* DH5α and derivative strains were grown in Luria-Bertani broth (LB) or on Luria-Bertani agar (LA) at 37°C supplemented with the appropriate antibiotics. Antibiotic concentrations used for *E. coli* strains were as follows: kanamycin (kan): 50 µg/ml, hygromycin (hyg): 200 µg/ml and antibiotic concentrations used for *M. smegmatis* and Mtb strains were as follows: kan: 25 µg/ml, hyg: 50 µg/ml, amoxicillin (20 µg/ml) and clavulanate (10 µg/ml). *M. smegmatis* mc^2^155 (Snapper et al., 1990), Mtb H37Rv and derivative strains were grown at 37°C in Middlebrook 7H9 broth supplemented with 0.2% glucose, 0.085% NaCl (for *M. smegmatis*), Middlebrook OADC enrichment (for Mtb), 0.2% glycerol (for *M. smegmatis*), 0.5% glycerol (for Mtb) and 0.05% Tween-80 or on Middlebrook 7H10/7H11 agar supplemented with 0.5% glycerol, 0.2% glucose, 0.085% NaCl or Middlebrook OADC enrichment (for Mtb). Liquid cultures were grown at 37°C with shaking at 100 rpm for *M. smegmatis* and 200 rpm for *E.coli*.

### 2.2 Genetic manipulation

The *M. smegmatis* mc^2^155 MurT-GatD CRISPRi dCas9 based depletion strain was constructed as previously described [the PAM sequence and sgRNA sequence primers are listed in **Table A3** (Rock et al., 2017)]. For protein localization studies, MurT-rseGFP was constructed by cloning wildtype *murT* (MSMEG_6276) with its 100 bp upstream region into a previously generated plasmid pMV306H-rseGFP (**Table A2**). Briefly, the MSMEG_6276 gene was PCR amplified and cloned into plasmid pMV306H-rseGFP for C-terminal tagging to generate plasmid pMurT-rseGFP, which was then electroporated into wildtype *M. smegmatis* mc^2^155. For electroporation of plasmid DNA, 1 μg of plasmid DNA was used to transform electro-competent mycobacterial cells or derivative strains by mixing 300 μl of cells with plasmid DNA and transferring the mixture into a pre-chilled 0.2 cm electroporation cuvette (Bio-Rad laboratories). The cells were incubated on ice for 15 minutes and the Bio-Rad Gene Pulser XCell™ (Bio-Rad laboratories) system was used to perform plasmid DNA electroporations with the following parameters: voltage: 2500 V, time constant: 25 μF, resistance: 1000 Ω and distance: 0.2 cm. After electroporation, 800 μl of LB media was added to the electroporation cuvette and the cells were transferred into a clean tube and incubated at 37°C overnight. Thereafter, the cells were pelleted by centrifugation at 12470 *Xg* and the supernatant was discarded followed by the resuspension of the cells in 500 μl of Middlebrook 7H9 broth. The cells were inoculated onto Middlebrook 7H10 agar containing appropriate antibiotics and incubated at 37°C for 3-5 days (Gordhan and Parish, 2001). The mRFP-GatD fusion was constructed by cloning wildtype *gatD* (MSMEG_6277) into a previously generated plasmid pTweety-mRFP (Stover et al., 1991). The MSMEG_6277 gene was PCR amplified and cloned into plasmid pTweety-mRFP for N-terminal tagging to generate plasmid pmRFP-GatD, which was electroporated as indicated above into *M. smegmatis* carrying the pMurT-rseGFP. For PknB-FLAG, the *pknB* (MSMEG_0028) gene was PCR amplified and cloned into plasmid pFLAGEM (Narrandes et al., 2015) derived from plasmid pSE100 (Guo et al., 2007) and electroporated as indicated above into *M. smegmatis* mc^2^155.

### 2.3 Quantitative real-time PCR

RNA was extracted using the Nucleospin RNA II kit (Machery Nagel) as per manufacturer’s instructions from cells grown at 37°C to an OD_600nm_ of 0.6 in 50 ml 7H9 broth. ATc or antibiotic supplementation of media where required was performed at the start of the culture. RNA was quantified using the Nanodrop. Prior to reverse transcriptase (RT)-PCR, 1 µg of RNA was treated with 1 µl TURBO DNaseI (Life Technologies) for 30 min at 37°C to remove residual genomic DNA. DNaseI was inactivated by the addition of 7 µl DNaseI removal resin followed by centrifugation for 5 min at 12470 *Xg*. A 23 µl aliquot was used for RT-PCR. A 2.5 µM reverse primer mix containing MSMEG_6276 (*murT*), MSMEG_6277 (*gatD*) and MSMEG_2758 (*sigA*) reverse primers (MurTRev: atgagcacgtcgtcacagtt, GatDRev: cgcggtggttttcgaatcc and SigARev: gggcgtgatgtccatctcct) was prepared and 2 µl of this mixture was added to TURBO DNase treated RNA. The housekeeping *sigA* gene allows for normalization because the gene is expressed at a consistent level throughout all growth phases. PCR reactions were performed using the following parameters: 94°C for 90 secs, 65°C for 3 min and 57°C for 3 min followed by incubation on ice. RT reactions were carried out using SuperScript III reverse transcriptase (Invitrogen) as per manufacturer’s instructions. Briefly, a 25 µl RT-positive reaction was set up using 12.5 µl of the annealed product, 4 µl of 25 mM MgCl_2_, 5 µl of 5× First Strand Buffer, 2 µl of 0.1 M DTT, 1 µl of 10 mM dNTPs and 0.8 µl of SuperScript III. The RT-negative reaction was set up with nuclease-free water replacing SuperScript III in the reaction. cDNA synthesis was carried out using the following parameters: 50°C for 50 min and 85°C for 5 min. The resulting cDNA was used for quantitative PCR (qPCR) performed using Sso Fast Evagreen Supermix (BioRad) as per manufacturer’s instructions. Briefly, 20 µl reactions were set up, each containing 10 µl Evagreen Supermix, 0.75 µl forward primer (MurTFwd: gaagtcgacgagatgcatgtg, GatDFwd: cgggcactggtacgagac) (10 µM), 0.75 µl reverse primer (MurTRev: atgagcacgtcgtcacagtt, GatDRev: cgcggtggttttcgaatcc) (10 µM), 2 µl cDNA and nucleasefree water. All reactions were incubated in the CFX96 Real-Time PCR detection system (BioRad) using the following parameters: 98°C for 2 min followed by 39 cycles consisting of three steps – 98°C for 5 sec, 60°C for 5 sec and 72°C for 5 sec with SYBR Green quantification at the end of each cycle. Melt curve analysis was conducted from 65°C with a gradual increase in 0.5°C increments every 0.05 sec to 95°C with SYBR Green quantification conducted continuously throughout this stage. The raw data was analyzed using the Biorad CFX Manager 3.0 Software (BioRad). Three independent biological replicates were assessed.

### 2.4 Cell envelope labeling

Fluorogenic probes used in this study include (i) Tetramethylrhodamine [TAMRA]-L-Ala-D-Gln-L-Lys-D-Ala [TetraFI-1]; (ii) non-amidated TAMRA-L-Ala-D-Glu-L-Lys-D-Ala [TetraFI-2]), (iii) TAMRA-D-Alanine (TADA), (iv) HCC-D-Alanine (HADA), (v) Alkyne-D-Alanine-D-Alanine (AlkDADA) and (vi) BODIPY-FL vancomycin. *M. smegmatis* mc^2^155 derivative strains grown to exponential phase were labelled with 500 µM TAMRA-L-Ala-D-Gln-L-Lys-D-Ala, TAMRA-L-Ala-D-Glu-L-Lys-D-Ala, TADA or HADA and analyzed for probe incorporation by fluorescence microscopy and time-lapse fluorescence microscopy. *M. smegmatis* mc^2^155 derivative strains grown to exponential phase were labelled with 2 mM AlkDADA and the probe was detected by click chemistry as previously described (Liechti et al., 2014). Briefly, 1 mM AlkDADA was added to cells grown to an OD_600nm_ of 0.6 and collected by centrifugation at 12 470 *Xg* followed by resuspension in 2 ml of Middlebrook 7H9 broth. The cells were then incubated for 2 hours at 37°C with shaking at 100 rpm. Subsequently, the cells were washed three times with PBSTB buffer (1X PBS, 0.05% BSA and 0.01% Tween20) and fixed for 10 minutes in 1 ml of 2% formaldehyde diluted in 1X PBS. Thereafter, the cells were centrifuged at 12 470 x*g* for 5 ml and the supernatant was discarded. The click chemistry reaction was conducted as follows: 920 µl of PBSTB buffer was used to resuspend the cells and 20 µl of CuSO_4_ (50 mM), 20 µl of TBTA (tris-(benzyltriazolylmethyl) amine) (6.4 mM), 20 µl of sodium ascorbate (60 mM) and 20 µl of an Azido probe (1 mM diluted in DMSO) were added, in the order stated, to the cells and mixed gently. The cells were further incubated at room temperature for 45 minutes and subsequently washed three times with 1X PBS. Thereafter, 5 µl of the cells was spotted onto glass slides which were visualized with the Nikon TI20 fluorescence microscope and the images taken were analyzed with the Fiji software (ImageJ version 1.47n [NIH]). Any image manipulation (brightness, contrast etc.) was applied to the entire image equally. Exponential-phase cultures of *M. smegmatis* mc^2^155 derivative strains were independently labelled with (100 µg/ml) BODIPY-FL vancomycin for 3 hours, which was subsequently washed out, and analyzed by flow cytometry and fluorescence microscopy for probe incorporation. Three independent biological repeats were assessed.

### 2.5 Time-lapse microscopy

*M. smegmatis* mc^2^155 derivative strains were grown in microfluidic devices as previously described (Dhar and Manina, 2015). Briefly, the bacterial cultures were grown to exponential phase in culture flasks at 37°C. Clumps of bacteria were removed by concentrating the cells and filtering through a 5 μm filter. The bacteria were seeded into a microfluidic device and imaged on a Delta vision Personal DV imaging system (GE HealthCare Life Sciences) using a 100x objective (Olympus Plan Semi Apochromat, 1.3 NA). The images were acquired at 10 min intervals using a CoolSnap HQ2 camera. Middlebrook 7H9 medium with or without 200 ng/mL Anhydrotetracycline (ATc+) and supplemented with FDAAs (labeling for 30 min) was circulated through the device at a flow rate of 25 μl/min. Images were analyzed and processed using Softworx (Version 4.1; Applied Precision, GE HealthCare) or ImageJ (Version 1.47n; NIH). Three independent biological repeats were assessed.

### 2.6 Ethidium bromide diffusion assay to monitor cell permeability

*M. smegmatis* mc^2^155 derivative strains were grown in 50 mL 7H9 medium at 37°C to an OD_600nm_ = 0.6. Cultures were centrifuged at 4500 rpm for 10 minutes and the supernatant was discarded. The pellet was then washed in 1X PBS. Thereafter, the pellet was resuspended in 7H9 containing 0.4% glucose and the OD_600nm_ was adjusted to 0.4. A 95 µl aliquot of bacterial suspension was placed into flat bottom, black 96-well plates (Thermo Scientific), followed by addition of ethidium bromide to final concentration of 10 µg/mL. Fluorescence was immediately measured with an excitation and emission wavelength of 530 nm and 585 nm respectively, every 60 seconds for 60 minutes at 37°C. Three independent biological repeats were assessed.

### 2.7 Lysozyme minimum inhibitory concentration determination

To determine the lysozyme MIC, cells were grown overnight at 37°C to an OD_600nm_ of 0.6 in 7H9 medium. Cultures were diluted 1:20 in 7H9 medium. Thereafter, a two-fold dilution series was setup for each strain in a clear, 96-well, round-bottom plate as follows: 50 μl of 7H9 broth was aliquoted into each well, except for the top and bottom rows of 8 wells which served as vehicle-plus-media only control and media only control wells. Lysozyme was added to the first column, starting at 2.5 mg/ml in a final volume of 100 μl and then diluted two-fold from the first column (column 1) to the last column (column 12). For the last column, 50 μl was discarded, so that all wells contained a final volume of 50 μl. After dilution of lysozyme and controls (media only or vehicle-plus-media), 50 μl of cells were added to each well. The plates were sealed and incubated at 37°C. After incubating plates at 37°C for 3 days, 15 μl of alamarBlue™ cell viability reagent (Invitrogen) was added to each well and further incubated at 37°C for 3 hours). Cell viability, and subsequent determination of MIC, was measured by assessing the fluorescence of reduced alamarBlue™ in a plate reader (excitation and emission at 530–560 and 590 nm, respectively). Three independent biological repeats were assessed.

### 2.8 Western blotting

For analysis of the phosphorylation status of PknB-FLAG in CRISPRi modified strains, cytoplasmic and membrane fractions of cell lysates from MurT-GatD depleted cells grown in media supplemented with a range of ATc concentrations (0 ng/ml-500 ng/ml) were separated on SDS-PAGE gels prepared with the TGX FastCast Acrylamide kit (BioRad), as per manufacturer’s instructions. Proteins were transferred to a Immune-Blot PVDF membrane (BioRad) using a Trans-Blot turbo transfer system (BioRad), as per manufacturer’s instructions. For immunological detection, the membrane was first washed three times with Tris buffered saline with Tween 20 (TBST) for 5 min each. The membrane was then incubated for 1 hour in blocking solution containing a primary monoclonal anti-FLAG M2 (α-FLAG) antibody (Sigma-Aldrich) at a final concentration of 10 µg/ml or an anti-phosphothreonine-peroxidase (α-P-Thr) antibody (Sigma-Aldrich) at a dilution of 1:50. Thereafter, the membrane was washed three times with TBST for 5 min each. For detection of the primary monoclonal anti-FLAG M2, the membrane was then incubated in blocking solution containing the secondary antibody, rabbit anti-mouse IgG-peroxidase antibody (Sigma-Aldrich) at a dilution of 1:80000. This was followed by three TBST washes for 5 min each. The membrane was transferred to a hybridization bag, treated with a chemiluminescent peroxidase substrate (CPS) and incubated at room temperature for 5 – 10 min. All excess substrate was removed and the membrane was exposed to X-ray film for 30– 60 secs. The X-ray films were developed in a dark room manually using the Axim developing and fixing solutions (Axim X-Ray industrial and medical).

### 2.9 Peptidoglycan extraction and labelling with an amide reactive dye

PG was extracted as previously described (Senzani et al., 2017). Briefly, wildtype *M. smegmatis* mc^2^155 was grown to OD_600nm_ of 2 and the cells were then harvested by centrifugation at 3500 *X*g for 10 min and resuspended in phosphate-buffered saline (PBS, pH 7.2). The cells were then lysed with a French press (Constant Systems). Insoluble material was obtained by centrifugation at 4000 *X*g for 30 min. The pellet was then resuspended in PBS containing 2% SDS and incubated at room temperature for 1 hour, then in PBS containing 2 mg/ml proteinase K and 2% SDS at 37°C for 24 hours and finally in PBS containing 2% SDS at 90°C for 1 hour. The extracted cell wall material was lyophilized, weighed and a 100 µg/ml of lyophilized cell wall material was resuspended in PBS and digested with 0.1 mg/ml mutanolysin for 24 hours. The digested material was harvested at 13000 *Xg* for 3 min, washed thrice with PBS. The pellet was resuspended in 500 µl PBS and labelled with 100 µg/ml of Alexa Fluor 488 NHS Ester (Sigma-Aldrich) for 3 hours.). The CytoFLEX flow cytometer (Beckman Coulter) was used for analysis of the labelled PG samples (100 µl per sample) in the FITC channel (excitation/emission maxima =494/517 nm). Three independent biological repeats were assessed.

### 2.10 Flow cytometric analysis of stained mycobacterial cells

Flow cytometry was used to analyze the labelling pattern of wildtype *M. smegmatis* mc^2^155, Mtb H37Rv or derivative genetically modified strains. Briefly, cells were grown in 5 ml of Middlebrook 7H9 broth supplemented with the appropriate antibiotics at 37°C with shaking at 100 rpm to an OD_600nm_ of 0.6. Thereafter, 200 µl of labelled cell cultures were transferred to a flat bottom, transparent 96 well plate (Thermo Scientific). The CytoFLEX flow cytometer (Beckman Coulter) was used for analysis of the labelled cells by detecting TAMRA signal in the PE-A channel (excitation/emission maxima = 546/579 nm) or the BODIPY-FL signal in the FITC channel (excitation/emission maxima =502/511 nm). The gain for the PE-A channel was reduced from 370 to 120 for all samples tested. Three independent biological repeats were assessed.

## 3 Results

### 3.1 Mycobacteria amidate the D-glutamate residues of PG for PG cross-linking

Recently, the mycobacterial MurT-GatD complex was identified and characterized (Maitra et al., 2021; Silveiro et al., 2023). In Mtb, the genes Rv3712-Rv3713 encode for the mycobacterial MurT and GatD homologs, respectively. Rv3712 and Rv3713 were predicted to be essential for *in vitro* growth of Mtb by Himar1 transposon mutagenesis and sequencing (TnSeq), are highly conserved among mycobacteria and physically interact to form an enzyme complex required for amidation of the D-Glu residues of PG for PG cross-linking (Sassetti et al., 2003; Maitra et al., 2021). To confirm that mycobacteria require PG amidation for cross-linking *in vivo*, a pair of fluorescent PG stem peptide mimics bearing either D-Gln or D-Glu at position 2 of the tetrapeptide probe [TetraFl-1 and TetraFl-2, respectively (**Figures 1B, C**), which report on the activity of LDTs (Pidgeon et al., 2019)] were used. We hypothesized that the lack of amidation in the D-Glu probe at position 2 would preclude stable incorporation into mycobacterial PG. As expected, D-Gln bearing tetrapeptide probes labelled *M. smegmatis* and Mtb cells, as assessed by microscopy and flow cytometry, while the D-Glu carrying tetrapeptide probe was not incorporated (**Figures 1A-C**). These data confirmed that for *in vivo* PG cross-linking in mycobacteria, prior D-Glu amidation of PG precursors by the MurT-GatD complex is required.

### 3.2 MurT-rseGFP and mRFP-GatD co-localize at sites of PG biosynthesis

**Figure 2:**
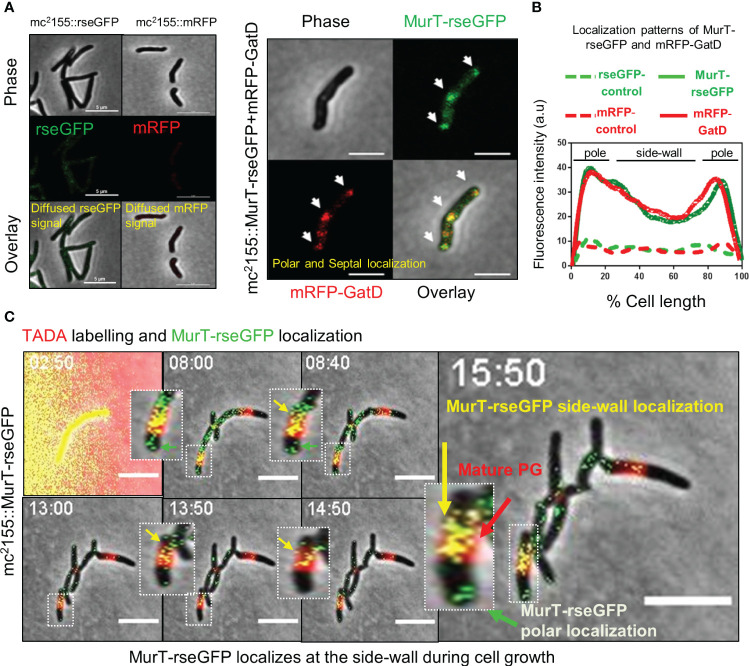
MurT-rseGFP and mRFP-GatD co-localize at cell poles and MurT-rseGFP co-localizes with mature PG. **(A)** Micrographs showing MurT-rseGFP and mRFP-GatD localization at the cell poles and septum. Also shown are the rseGFP and mRFP control strains displaying diffused rseGFP and mRFP signals. **(B)** Graph showing the localization patterns of MurT-rseGFP and mRFP-GatD in *M. smegmatis* mc^2^155. A hundred cells (n=100) were analyzed for each strain and the lines represent the average of the fluorescence intensity across the cells. **(C)** Time-lapse microscopic analysis of MurT-rseGFP in cells labelled with TADA (shown by red arrow) grown in 7H9 media supplemented with hygromycin, kanamycin and TADA (labeling performed for 30 min). MurT-rseGFP localizes at the cell poles (green arrows) and also with maturing PG (yellow arrows). Time-stamp is in hours. Scale bar is 5 µm.

We next investigated whether the MurT-GatD enzymes associated with PG amidation spatially localize to areas of new PG synthesis. Mycobacteria are known to spatially localize the major cell wall biosynthesis processes to the cell poles during cell extension (Aldridge et al., 2012; Botella et al., 2017b). In mycobacteria, the tropomyosin-like protein, DivIVA, which directs polar growth interacts with enzymes which facilitate early steps of cell wall biosynthesis at the cell poles forming a complex of enzymes termed the elongosome, which facilitates polar cell wall synthesis (Meniche et al., 2014). To investigate whether the MurT-GatD complex also traffics to these locales in mycobacteria, we constructed a merodiploid *M. smegmatis* strain, mc^2^155::MurT-rseGFP+mRFP-GatD, wherein the native *murT* gene (MSMEG_6276) was fused to rseGFP and the native *gatD* (MSMEG_6277) was fused to mRFP. These were introduced at the *attB* and *t-RNA* glycine phage integration sites in the genome, respectively. *M. smegmatis* strains carrying untagged rseGFP and mRFP alleles (mc^2^155::rseGFP and mc^2^155::mRFP) were also generated in a similar manner to serve as controls. Microscopic analysis of the control strains revealed diffuse and low rseGFP and mRFP signals (**Figure 2A**); whereas microscopic analysis of mc^2^155::MurT-rseGFP+mRFP-GatD revealed that MurT-rseGFP and mRFP-GatD co-localize at the cell poles and the septum, in addition a minimal co-localization signal was also observed at the side-wall (**Figures 2A, B**).

### 3.3 MurT-rseGFP co-localizes with maturing PG during cell growth

As recent studies suggest that mycobacteria remodel side-wall PG into mature 3-3 cross-linked PG and also repair the side-wall PG when exposed to cell wall damaging agents (Baranowski et al., 2018; García-Heredia et al., 2018), and as we also observed a signal of MurT-rseGFP and mRFP-GatD co-localization at the side-wall, we hypothesized that incorporation of PG precursors into the side-wall for PG remodelling would also require prior amidation by the MurT-GatD complex. To investigate this, spatio-temporal localization of MurT-rseGFP during cell growth was monitored by time-lapse microscopy of MurT-rseGFP cells labelled with a FDAA probe (TADA) during growth (**Figure 2C**, **Supplementary Figure S1** and **movies S1**, **S2**). MurT-rseGFP co-localized with TADA labelling of the cell poles. As the cells expanded, the TADA label (red) was detected at the side-wall (representing mature PG) and the MurT-rseGFP (green) fluorescence signal was observed at the cell poles and also at the side-wall, co-localizing (yellow signal) with the TADA label representing maturing PG (**Figure 2C**, **Supplementary Figure S1B** and **movies S1**, **S2**). The resulting yellow signal (i.e. a mixture of MurT-rseGFP [green] and TADA signal [red]) suggested that PG remodelling at the side-wall also features cytoplasmic MurT-GatD facilitated PG precursor amidation.

### 3.4 MurT-GatD are essential for growth

**Figure 3:**
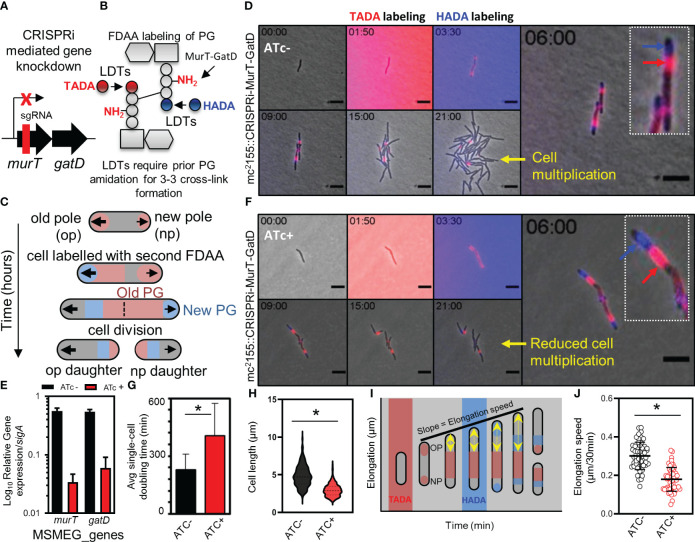
Depletion of MurT and GatD reduces cell growth. **(A)** Schematic representation of the binding region of the sgRNA used for CRISPRi mediated repression of the *murT*-*gatD* operon. **(B)** Schematic representation of MurT-GatD mediated PG precursor amidation. **(C)** Schematic representation of labelling of mycobacterial cells with FDAAs for analysis of cell growth. **(D)** Time-lapse microscopy analysis of *M. smegmatis* CRISPRi-MurT-GatD cells without CRISPRi activation. **(E)** qPCR analysis of *murT* and *gatD* expression during normal conditions or during gene knockdown conditions. Shown are the *murT* and *gatD* transcript levels normalized to *sigA*. **(F)** Time-lapse microscopy analysis of *M. smegmatis* CRISPRi-MurT-GatD cells with CRISPRi activation. **(G)** The average single-cell doubling time of cells expressing or not expressing MurT and GatD analysed by time-lapse microscopy. Depletion of MurT and GatD increases the doubling time of the cells. **(H)** Violin plots showing the cell length of cells expressing MurT-GatD compared to cells depleted of MurT-GatD (n=300 per experiment). **(I)** Schematic representation of how cell elongation was measured in FDAA labelled cells expressing or not expressing MurT and GatD with the nearest FDAA signal to the old cell pole as fiducial marker for cell pole elongation over-time. **(J)** Scatter plot showing the polar elongation speed of cells expressing or not expressing MurT and GatD (n=100 cells for each experiment. Statistical analysis was conducted using Student t-test. *: p-value <0.05. sgRNA – short guide RNA. Scale bar is 5 µm.

Next, we attempted to delete *murT* from the genome of wildtype *M. smegmatis* mc^2^155 to confirm the essentiality of this enzyme for mycobacterial viability as previously suggested (De Wet et al., 2020). Deletion of *murT* was only achieved in the presence of a second copy of *murT* expressed from a *tetO* promoter, introduced into *M. smegmatis* mc^2^155 by an episomal shuttle vector (**Supplementary Figure S2**). This was consistent with prior reports predicting essentiality of *murT* and *gatD* (Dragset et al., 2019). To assess the role of the MurT-GatD complex in growth of *M. smegmatis* mc^2^155, a CRISPRi based repression system was used to titrate levels of *murT* and *gatD* (**Figure 3A**). The experiment was conducted in cells labelled with fluorescent D-amino acids (FDAAs) which are incorporated into existing PG by LDTs during PG remodeling (**Figure 3B**), and this allowed us to study the dynamics of PG remodelling during MurT-GatD depletion. A previously reported highly effective short-guide RNA (sg-RNA; 5’-GAGCTGATCACGCGACAGAT-3’) (De Wet et al., 2020), targeting the 5’ region of *murT* was used as a guide for CRISPRi dCas9 mediated inhibition of *murT*-*gatD* transcription. Sequential labelling, first with TADA, followed by HADA enabled the study of PG remodelling dynamics (**Figure 3C**). Using this approach with mc^2^155::CRISPRi-MurT-GatD cells without CRISPRi induction (i.e. ATc-) showed polar incorporation of new cell wall material as previously demonstrated (Aldridge et al., 2012), with mature cell wall material localizing to the lateral axis (**Figures 3C, D**, **Supplementary Figure S3** and **movies S3**-**S6**). To assess the requirement for MurT-GatD in PG biosynthesis, the CRISPRi platform in mc^2^155::CRISPRi-MurT-GatD was induced (with ATc) to deplete the transcripts encoding these enzymes. A significant reduction in transcripts was detected using qRT-PCR (**Figure 3E**). Depletion of MurT and GatD resulted in reduction of growth, with cells being shorter relative to cells expressing these enzymes (**Figures 3F-H**, **Supplementary Figure S4** and **movies S7**-**S10**). However, the FDAA labelling pattern did not significantly differ upon gene depletion. Considering this, we next investigated polar elongation speed by measuring the region of unlabelled polar extension over time after labelling with FDAA, with the point of previous FDAA labelling used as a fiducial marker (**Figure 3I**). Cells depleted of MurT-GatD displayed a significantly reduced polar elongation speed, compared to cells replete with these enzymes, pointing to a role for PG amidation in regulation of cell elongation (**Figure 3J**).

### 3.5 PG cross-linking in mycobacteria requires prior amidation of D-glutamate in lipid II

**Figure 4:**
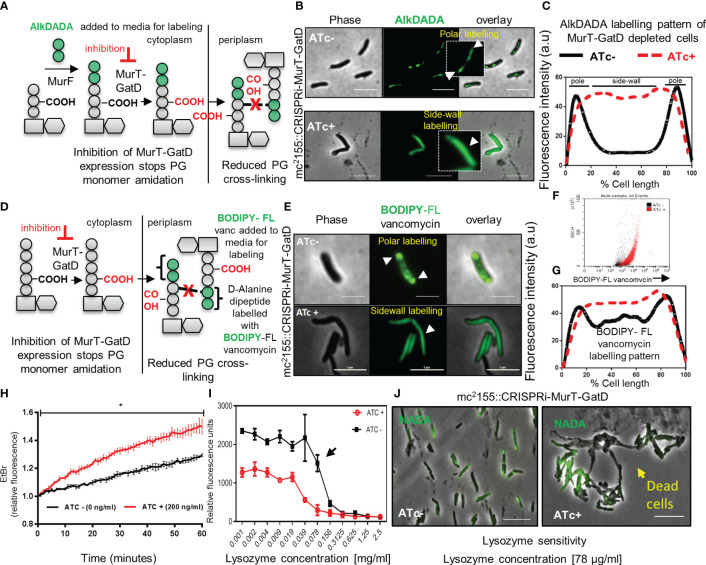
MurT-GatD depletion causes perturbed PG remodelling, increased cell wall permeability and increased sensitivity to lysozyme. **(A)** Schematic representation of cell wall labeling with AlkDADA showing incorporation of the label into PG precursors during *de novo* PG biosynthesis. **(B)** Micrographs of *M. smegmatis* CRISPRi MurT-GatD cells expressing or not expressing MurT-GatD. Inhibition of MurT-GatD expression causes increased side-wall labeling with AlkDADA. **(C)** Graph showing the AlkDADA labeling pattern of cells depleted of MurT-GatD compared to MurT-GatD expressing cells. A hundred cells (n=100) were analyzed for each strain and the lines represent the average of the fluorescence intensity across the cells. Cells with clear increased sidewall signal of AlkDADA were counted *vs* polar and/or septal labeling in cells not exposed to ATc. **(D)** Schematic representation of cell wall labeling with BODIPY-FL vancomycin showing labeling of PG precursors in the periplasm. **(E)** Micrographs of BODIPY-FL vancomycin labeled MurT-GatD depleted cells in comparison with cells expressing the proteins. Inhibition of MurT-GatD expression causes increased side-wall labeling with BODIPY-FL vancomycin. n=100 labelled cells per experiment. **(F)** Flow cytometry quantification of BODIPY-FL vancomycin labelled MurT-GatD depleted cells in comparison to cells expressing the proteins. **(G)** Graph showing the BODIPY-FL vancomycin labeling pattern of cells depleted of MurT-GatD compared to MurT-GatD expressing cells. A hundred random cells (n=100) were analyzed for each strain and the lines represent the average of the fluorescence intensity across the cells. **(H)** Ethidium bromide uptake into cells depleted of MurT-GatD in comparison to cells expressing the proteins. **(I)** Sensitivity of MurT-GatD depleted cells to lysozyme. The black arrow indicates a two-fold change in susceptibility to lysozyme. **(J)** Micrographs of NADA (3-[(7-Nitro-2,1,3-benzoxadiazol-4-yl)amino]-D-alanine hydrochloride) labelled cells depleted of MurT-GatD and exposed to lysozyme (~80 µg/ml). Lysozyme exposure caused cell death in cells depleted of MurT-GatD. Statistical analysis was conducted using Student *t*-test. *:p-value < 0.0001. Scale bar is 5 µm.

As MurT-GatD depleted cells did not lyse or lose their inherent shape (**Figure 3F**), we sought to investigate whether depletion of MurT and GatD in mycobacterial cells would result in perturbed PG biosynthesis using an alkyne functionalized fluorescent dipeptide (D-Alanine-D-Alanine) probe AlkDADA (**Figure 4A**) (Liechti et al., 2014). AlkDADA is linked to a lipid II precursor during cell growth to form labelled lipid II which, if incorporated into the existing PG, can be detected on the cell surface by click chemistry (Liechti et al., 2014). As expected, cells expressing MurT-GatD displayed polar labelling with AlkDADA and reduced labeling at the side-wall indicative of insertion of the labelled PG precursors at the cell poles (**Figures 4B, C**). In contrast, depletion of MurT-GatD caused diffused labeling with AlkDADA in majority of cells analyzed indicative of reduced PG remodelling along the side-wall (**Figures 4B, C**). Side-wall PG remodelling involves the formation of 3-3 cross-links from 4-3 cross-links by LDTs (García-Heredia et al., 2018) and as a result, the D-Alanine-D-Alanine (DADA) dipeptide is removed from the PG. In Chlamydia, this compound has been shown to be primarily incorporated into lipid II (Liechti et al., 2014). Similarly, in mycobacteria, it has been shown that AlkDADA can be incorporated into lipid II (García-Heredia et al., 2018), although it is not yet clear if this is its primary mode of incorporation. Increased AlkDADA signal in MurT-GatD depleted cells is suggestive of perturbed PG remodelling in these cells. To confirm this observation, a fluorescent BODIPY-FL vancomycin probe, which binds the terminal D-Ala-D-Ala (DADA) moiety of the PG precursor, was used to probe PG remodelling in cells depleted of MurT and GatD (**Figure 4D**). Under replete conditions in the absence of ATc, the bacteria exhibited labeling of the poles and septum (**Figure 4E**). However, when the cells were depleted of MurT-GatD following the addition of ATc, the bacteria displayed increased levels of diffused labelling along the lateral axis of the cell wall. This observation was independently confirmed using flow cytometry, which revealed increased labeling of the cells with BODIPY-FL vancomycin as seen by microscopy (**Figures 4E-G**). As the terminal DADA moiety of PG precursors is used as a substrate for cross-linking by PBPs or can be cleaved by D,D-carboxypeptidases during PG remodelling (Vollmer et al., 2008), the increased labelling with BODIPY-FL vancomycin along the side-wall suggests that PG remodelling is decreased when MurT and GatD are depleted or reflects reduced PG remodelling dynamics by D,D-carboxypeptidases or increased cell wall permeability (Ngadjeua et al., 2018).

To assess whether the reduced PG-cross-linking in MurT-GatD depleted cells is associated with changes in cell wall permeability, we used an ethidium bromide diffusion assay as previously described (Senzani et al., 2017). Cells depleted of MurT-GatD exhibited increased cell wall permeability, evidenced by increased ethidium bromide fluorescence (**Figure 4H**). We hypothesized that reduced PG cross-linking in MurT-GatD depleted cells would simultaneously cause an increased sensitivity to PG targeting antimicrobials such as lysozyme. A lysozyme minimum inhibitory concentration experiment revealed that MurT-GatD depleted cells were indeed sensitive to killing by lysozyme and microscopic analysis of these cells showed that lysis occurred following exposure while cells expressing MurT-GatD displayed less sensitivity to lysozyme exposure (**Figures 4I, J**). Furthermore, to assess the level of PG amidation in MurT-GatD depleted cells, the PG of lysed MurT-GatD- expressing and depleted cells was extracted and labelled with an amide reactive dye, Alexa Fluor 488 NHS Ester which labels primary amines (R-NH_2_). The labelled PG from MurT depleted cells contained a lower dye signal, suggestive of a decrease in the level of PG amidation (**Supplementary Figure S5**).

### 3.6 PknB regulates cell growth in response to PG amidation

**Figure 5:**
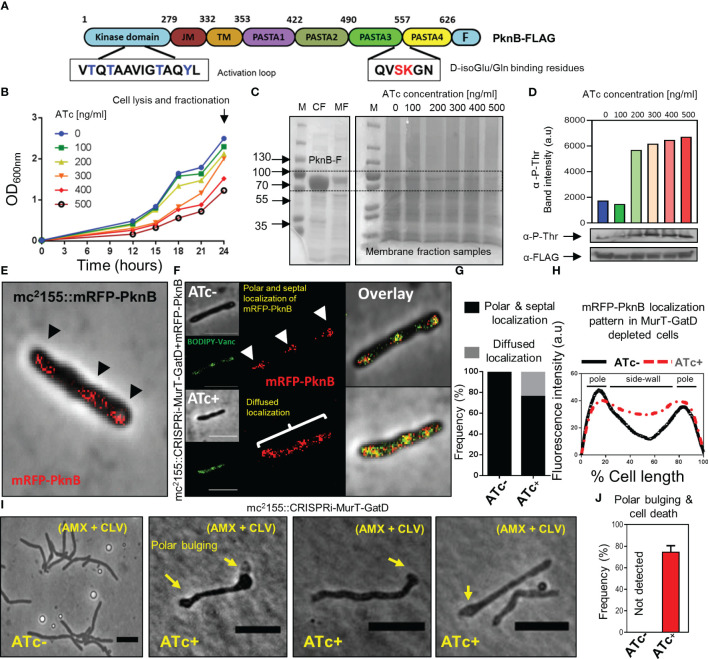
Reduced MurT-GatD activity affects PknB activity. **(A)** Schematic representation of the domain structure of PknB and shown are the D-Glu binding residues (red font) and the activation loop with threonine residues (blue font) subject to phosphorylation upon PknB activation. **(B)** Growth curve of *M. smegmatis* mc^2^155::CRISPRi-MurT-GatD+PknB-FLAG grown in media supplemented with 100-500 ng/ml of ATc. **(C)** SDS-page gels showing expression of PknB-FLAG in the cytoplasmic fraction (CF) and presence of PknB-FLAG also in the membrane fraction (MF). MFs from cells depleted of MurT-GatD were used to assess PknB phosphorylation. **(D)** Western blot analysis of PknB-FLAG phosphorylation from MFs of MurT-GatD depleted cells. MurT-GatD depletion causes increased phosphorylation of PknB. **(E)** mRFP-PknB localizes at the cell poles and cell septum (black arrow heads). **(F)** Depletion of MurT and GatD causes diffused mRFP-PknB localization. **(G)** Histogram showing the frequency of cells showing polar and septal or diffused mRFP-PknB localization (n=100). Cells with a diffused signal of mRFP-PknB were counted *vs* polar and/or septal localization pattern in cells not exposed to ATc. **(H)** Graph showing the mRFP-PknB localization pattern in cells depleted of MurT-GatD compared to MurT-GatD expressing cells. A hundred random cells were analysed per experimental condition (i.e. ATc- *vs* ATc+) and the lines represent the average of the fluorescence intensity across the cells. **(I)** Time-lapse micrographs of MurT-GatD expressing and depleted cells exposed to sub-lethal concentration of amxocillin (20 µg/ml) and clavulanate (10 µg/ml). **(J)** Histogram showing the frequency of dead cells with polar bulges, 25 time-lapse micrographs were analysed for each experiment. Scale bar is 5 µm.

PknB-like kinases have been implicated in the regulation of PG biosynthesis in most Gram-positive bacteria (Pereira et al., 2011). In mycobacteria, uncross-linked PG is detected by PknB through C-terminal PASTA domains, leading to the autophosphorylation of the N-terminal kinase domain at threonine residues (**Figure 5A**) (Kaur et al., 2019). In mycobacteria, PknB is essential for cell growth (Sassetti et al., 2003; Dejesus et al., 2017) and it was previously demonstrated that its extracellular PASTA domains (PASTA3 and PASTA4) are required for localization at the cell poles and septum for regulation of PG biosynthesis and cell growth (Mir et al., 2011).

Recently, it was demonstrated that abrogation of D-Gln interacting residues (Ser_556_ and Lys_557_) of PknB, which specifically interact with the terminal carboxyl group of D-Gln, results in perturbation of PknB function and localization (Kaur et al., 2019). Considering this, we hypothesized that the reduced PG amidation resulting from depletion of MurT-GatD would consequently affect PknB function. To investigate this, phosphorylation of a FLAG-tagged PknB was assessed in *M. smegmatis* depleted of MurT and GatD, using a titration of the ATc inducer [100 ng/ml-500 ng/ml]. Here, we hypothesized that addition of a second copy of *pknB*, carrying the FLAG-tag, would not materially affect MurT-GatD function. As expected, the MurT-GatD depleted strain (carrying the FLAG-tagged PknB) displayed a ATc dose-dependent reduction in growth (**Figure 5B**). These cells were subsequently lysed for collection of membrane fractions to analyze PknB phosphorylation with an anti-phosphothreonine antibody. We first confirmed the expression of PknB-FLAG without CRISPRi induced repression of MurT-GatD (**Figure 5C**). An SDS page analysis of the samples confirmed the presence of a ~70kDa protein band in both the cytoplasmic fraction (CF) and the membrane fraction (MF) corresponding with the expected band size for *M. smegmatis* mc^2^155 PknB (66.2kDa). MurT-GatD depletion was associated with increased PknB phosphorylation, in a ATc dose dependent manner, as analysed by the band intensity of PknB detected by an anti-phosphothreonine antibody (**Figure 5D**). These results demonstrated that the lack of PG amidation results in PknB dysregulation, which likely affects the downstream activation of PknB substrates and signal for cell growth cessation.

### 3.7 PknB localization is perturbed in MurT-GatD depleted cells

Since PknB activity is increased in MurT-GatD depleted cells, we next sought to determine the localization of PknB in these cells. To assess this, wildtype PknB was fused to mRFP on the N-terminus and the construct introduced into both the *M. smegmatis* mc^2^155 and mc^2^155::CRISPRi-MurT-GatD strains. An N-terminal fusion was used to ensure no disruption of the functionality in the C-terminal PASTA domains. The mRFP-PknB localized to the cell poles and septum in mc^2^155::mRFP-PknB and mc^2^155::CRISPRi-MurT-GatD+mRFP-PknB without CRISPRi induction (i.e. ATc-) (**Figure 5E, F**). Upon CRISPRi induction, most of the cells analyzed displayed a similar mRFP-PknB localization pattern as seen in wildtype cells. However, mRFP-PknB also displayed diffused localization in a subpopulation of MurT-GatD depleted cells (**Figures 5E, G, H**), indicative of perturbed PknB function as observed *via* the analysis of PknB phosphorylation status.

### 3.8 MurT-GatD depletion causes sensitivity to β-lactam antibiotics

The importance of lipid II amidation for PG cross-linking in mycobacteria highlights a vulnerability that can be exploited for inhibition of mycobacterial growth using cell wall targeting antibiotics (Maitra et al., 2021). We hypothesized that increased cell wall permeability as a result of reduced levels of amidated lipid II for PG biosynthesis in the MurT-GatD depleted cells would cause increased sensitivity to β-lactam antibiotics and result in rapid killing of the bacteria. To investigate this, time-lapse microscopic analysis of the growth of the MurT-GatD depleted mc^2^155::CRISPRi-MurT-GatD cells in the presence of a sub-lethal concentration of amoxicillin (AMX) (20 µg/ml) and clavulanate (CLV) (10 µg/ml) was performed. CLV was included to inhibit β-lactamases as *M. smegmatis* expresses numerous potent β-lactamases (Flores et al., 2005). AMX-CLV treated MurT-GatD depleted cells were unable to elongate, formed bulges at the cell poles and ultimately lysed. In contrast, cells expressing MurT-GatD were less sensitive to killing by AMX+CLV (**Figures 5I, J**, **Supplementary Figure S6** and **movies S11**, **S12**). Collectively, these results demonstrate the importance of PG amidation for mycobacterial cell growth and highlight the MurT-GatD complex as potential drug targets for TB drug development.

## 4 Discussion

β-lactam antibiotics, such as penicillin, inhibit the critical cross-linking step of PG biosynthesis, facilitated by PBPs, and have shown great success for treatment of a variety of bacterial infections in the clinical setting (Hugonnet et al., 2009; Diacon et al., 2016). This suggests that the PG biosynthesis pathway and enzymes involved are vulnerable drug targets that can be exploited for the development of new and more effective antibiotics. To facilitate these efforts, fluorescent probes have been used extensively to study PG biosynthesis in live bacterial cells to elucidate previously unknown functions of PG-associated enzymes, thus enabling further identification of potential drug targets (Aldridge et al., 2012; Liechti et al., 2014; Botella et al., 2017a). *Via* the use of fluorescent PG stem peptide mimics with amidation modifications on the second amino acid (i.e. D-Gln instead of D-Glu) of the stem peptide which are substrates of LDTs, we confirmed that this modification is required for the PG cross-linking reaction. We further investigated the essentiality of this modification in PG biosynthesis using CRISPRi mediated depletion of the enzymes (the MurT-GatD complex) required for this process. It has been shown that MurT and GatD catalyze the amidation reaction of the D-Glu residue in the peptide stem of PG precursors in *S. aureus*, *S. pneumoniae* and mycobacteria (Münch et al., 2012; Liu et al., 2017; Morlot et al., 2018; Nöldeke et al., 2018; Maitra et al., 2021). However, the phenotypic defects associated with the loss of PG amidation due to inactivation of MurT-GatD in mycobacteria remained unclear.

MurT and GatD are arranged in a bicistronic operon, which is conserved in various bacterial species including mycobacteria. Deletion of the native *murT* gene using the two step allelic exchange mutagenesis technique was only possible in the presence of a second copy of the *murT* gene, thus confirming the essentiality of this operon. We further used a CRISPR interference based programmable transcriptional repression platform coupled with time-lapse microscopy to investigate the role of this enzyme complex for mycobacterial cell growth (Dhar and Manina, 2015; Rock et al., 2017). Time-lapse microscopic analysis of MurT and GatD depleted cells demonstrated that repression of the *murT*-*gatD* operon resulted in growth inhibition. It has been previously reported that PG transpeptidases (both D,D-transpeptidases [DDTs] and LDTs) required for PG cross-linking only recognize amidated PG precursors as substrates (Zapun et al., 2013; Pidgeon et al., 2019). For mycobacteria, it remains to be shown whether mycobacterial DDTs also require amidated PG precursors to form PG cross-links. However, inhibition of PG precursor amidation in mycobacteria is expected to inhibit PG cross-linking by LDTs. To investigate this, MurT-GatD depleted cells were labelled with various fluorescent PG probes (FDAAs and a vancomycin analogue – BODIPY-FL vancomycin) and PG remodelling dynamics were assessed. FDAA labelled MurT-GatD depleted cells did not show changes in PG biosynthesis dynamics relative to cells expressing these enzymes. This could be due to the fact that LDTs require only one of the stem peptides to be amidated (Zapun et al., 2013). Interestingly, BODIPY-FL vancomycin which binds non-cross-linked PG precursors displayed an abnormal labeling pattern in MurT-GatD depleted cells. The non-cross-linked PG precursors were detected all along the lateral axis of the cell surface (i.e. diffused staining) which suggested that PG cross-linking by PG transpeptidases is reduced in MurT-GatD depleted cells.

To further study the role of MurT and GatD in mycobacteria, protein localization experiments were performed with rseGFP and mRFP tagged MurT and GatD, respectively. We found that MurT-rseGFP and mRFP-GatD co-localized at the cell poles and cell division septum. In other bacterial species MurT interacts with GatD and activates GatD function (Münch et al., 2012; Morlot et al., 2018). In mycobacteria, both these proteins were previously shown to interact in a mycobacterial protein fragment complementation (MPFC) assay (Maitra et al., 2021), and also identified by proteomic analysis as part of proteins enriched in the plasma membrane domain of mycobacteria known as the intracellular membrane domain (IMD) localized at the cell poles, which contains enzymes associated with cell wall biosynthesis (Hayashi et al., 2016). Furthermore, a high throughput imaging screen of a library of fluorescently tagged *M. smegmatis* proteins revealed a cell pole localization pattern of MurT-Dendra (Zhu et al., 2021), consistent with the localization pattern we observed in our study. These findings indicate that the MurT-GatD complex also forms part of both the elongosome and divisome complexes, required for synthesis of the cell wall at the poles and the cell division septum, respectively.

**Figure 6:**
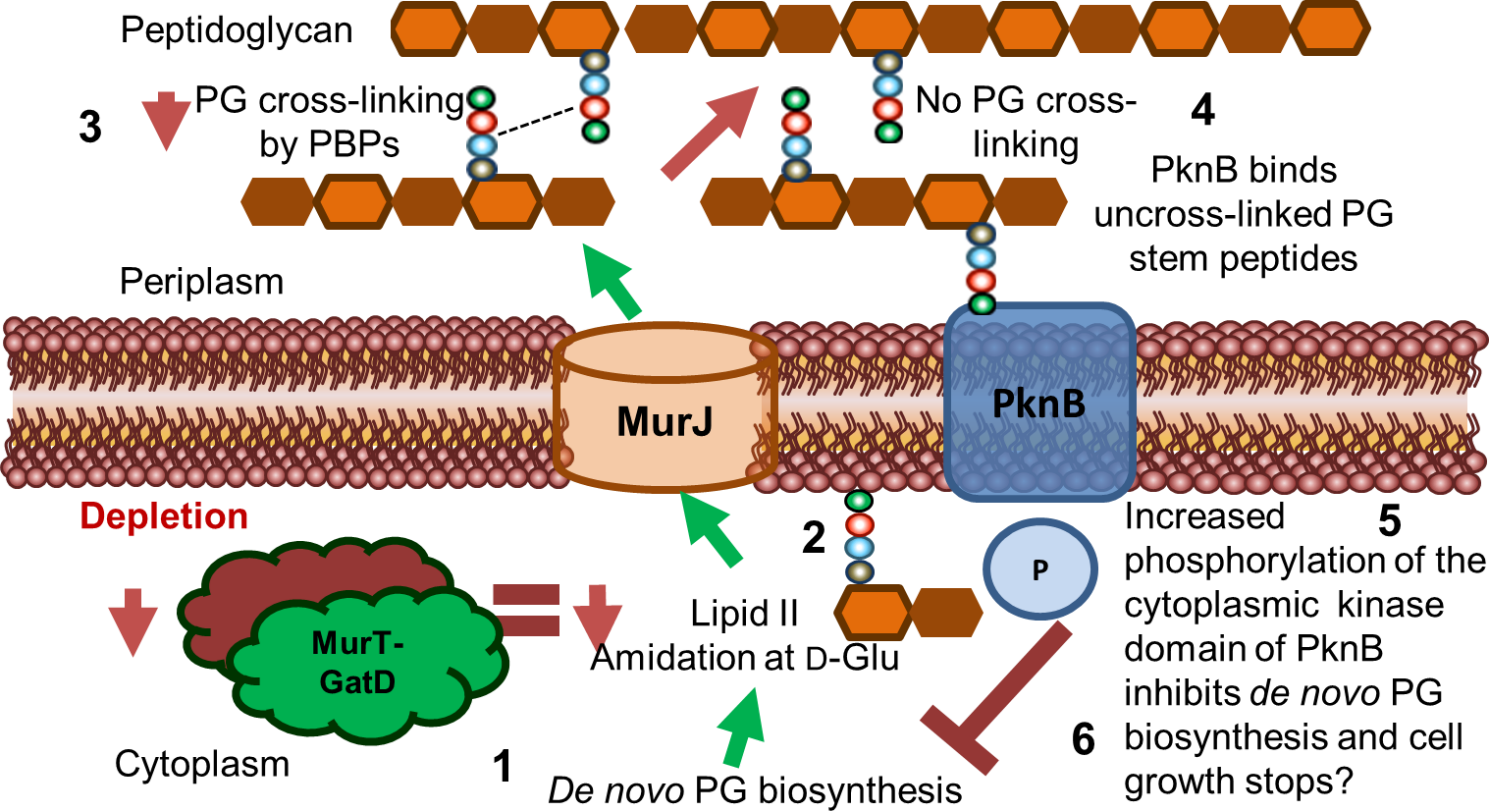
Proposed model for the role of MurT and GatD during PG biosynthesis and cell growth in mycobacteria. Lipid II biosynthesis involves MurT-GatD amidation of D-glutamate of the PG stem peptide. (1) Depletion of MurT-GatD causes decreased lipid II amidation at D-Glu (2) and non-amidated lipid II is not cross-linked into existing PG by PBPs (3). PknB binds uncross-linked PG stem peptides (4) and its cytoplasmic kinase domain auto-phosphorylates and causes inhibition of PG biosynthesis resulting in cessation of cell growth (6).

Recently, it was demonstrated that mycobacteria remodel the side-wall during growth (Baranowski et al., 2018); and Garcia-Heredia et al. showed that damage to the mycobacterial cell wall as a result of lysozyme exposure also requires side-wall remodeling and repair (Baranowski et al., 2018; García-Heredia et al., 2018). We found, using time-lapse microscopy, that MurT-rseGFP co-localized with TADA, a monopeptide FDAA probe, during cell growth. This suggested that MurT-GatD also localizes with maturing PG. We also hypothesized that if the PG is loosely cross-linked as a result of MurT-GatD depletion, the cell wall would be more permeable as previous studies showed that perturbations in PG remodelling causes increased cell wall permeability (Senzani et al., 2017). To investigate this, we used an ethidium bromide diffusion assay and showed that depletion of MurT and GatD was associated with increased cell wall permeability. This data demonstrates that amidation of PG plays a crucial role in PG biosynthesis and maturation in mycobacteria (**Figure 6**).

We further studied how PknB regulates PG biosynthesis in response to reduced PG cross-linking as a result of depletion of MurT-GatD in *M. smegmatis*. PknB is known to regulate PG biosynthesis by sensing PG cross-linking through extracellular PASTA domains which also facilitate the localization of PknB to the cell poles and septum (Mir et al., 2011). Specifically, residues that are located in a α-helix locker between PASTA3 and PASTA4 domains of PknB bind the carboxyl terminal of D-Gln/D-Glu and mDAP residues in the stem peptide of uncross-linked PG precursors and this causes autophosphorylation of the cytoplasmic kinase domain of PknB (Kaur et al., 2019). The activated PknB kinase domain regulates the function of PG biosynthesis enzymes including MurA, CwlM and MurJ through phosphorylation (Turapov et al., 2015; Turapov et al., 2018). We confirmed that depletion of MurT and GatD causes hyper-activation of PknB. Localization experiments of mRFP-PknB in MurT-GatD depleted cells also displayed perturbed mRFP-PknB localization indicative of changes in PknB function in these cells. We next hypothesized that cells depleted of MurT and GatD are highly dependent on PBPs to remain viable. To test this, we exposed the MurT-GatD depleted cells to a sub-lethal concentration of amoxicillin which inhibits the transpeptidase reaction of high molecular weight PBPS in the presence of clavulanate required for inhibition of β-lactamases (Hugonnet et al., 2009; Dhar et al., 2015; Diacon et al., 2016). Cells depleted of MurT and GatD were found to be highly sensitive to amoxicillin-clavulanate killing, showing reduced growth, polar swelling and eventual cell lysis. These data highlight the MurT-GatD complex as a vulnerable target for inhibition of PG biosynthesis.

Considering that MurT and GatD depletion resulted in reduced PG cross-linking and reduced growth, and that the mycobacterial serine/threonine protein kinase PknB is activated by uncross-linked PG precursors, we propose a model where PknB senses the level of uncross-linked PG as a result of the level of PG amidation caused by activity of MurT and GatD and regulates mycobacterial cell growth (**Figure 6**). Taken together, we offer evidence for the further development of MurT and/or GatD as novel drug targets for TB treatment.

## Data availability statement

The original contributions presented in the study are included in the article/**Supplementary Material**. Further inquiries can be directed to the corresponding author.

## Author contributions

MS and BK conceived the project. MS performed experiments, analyzed data and prepared the figures and manuscript. MP and MV provided fluorescent PG probes. ND assisted with experiment design and supervised the imaging experiments.
