# Supplementary material for: Amidation of glutamate residues in mycobacterial peptidoglycan is essential for cell wall cross-linking
Source: Front Cell Infect Microbiol. 2023 Aug 24;13:1205829. doi: 10.3389/fcimb.2023.1205829 (PMC10484409; doi:10.3389/fcimb.2023.1205829)
Supplement: Supplementary file 13 [file DataSheet_1.pdf]

Supplementary Material

Amidation of glutamate residues in mycobacterial peptidoglycan is essential for cell wall cross-linking

Moagi T. Shaku [1], Karl L. Ocius [2], Alexis J. Apostolos [2], Marcos Pires [2], Michael VanNieuwenhze [3], Neeraj Dhar [4,$,¶], and Bavesh D. Kana [1,*]

*** Correspondence:** Bavesh D. Kana: bavesh.kana@wits.ac.za

[1] DST/NRF Centre of Excellence for Biomedical TB Research, Faculty of Health Sciences, University of the Witwatersrand, National Health Laboratory Service, Johannesburg, 2001, South Africa

[2] Department of Chemistry, Lehigh University, Bethlehem, Pennsylvania 18015, USA

[3] Department of Chemistry, Indiana University Bloomington, 800 East Kirkwood Avenue , Bloomington, Indiana 47405, USA

[4] Global Health Institute, Ecole Polytechnique Fédérale de Lausanne, 1015 Lausanne, Switzerland

[$] Present address: Vaccine and Infectious Disease Organization, University of Saskatchewan, Saskatoon, SK S7N 5E3, Canada

[¶] Present address: Department of Biochemistry, Microbiology and Immunology, University of Saskatchewan, Saskatoon, SK S7N 5E5, Canada.

.

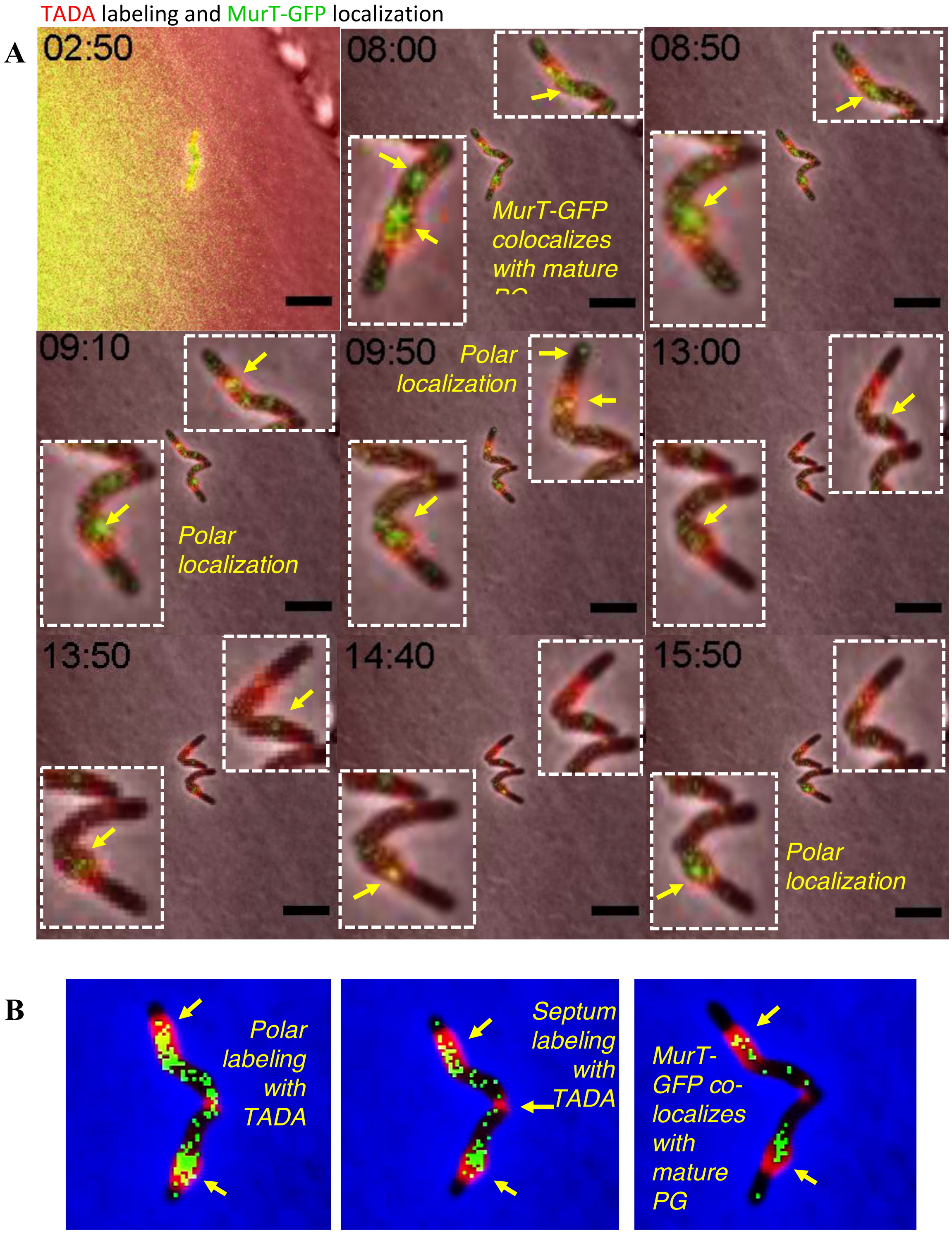

Supplementary Figure S1. Time-lapse microscopy analysis of TADA labelled MurT-GFP cells. (A) The MurT-GFP cells were grown first in media supplemented with 1 mM TADA for 15 min. The TADA supplemented media was washed out with no-label media and time-lapse microscopy of the TADA labelled MurT-GFP cells was performed for 24 hours**.** MurT-GFP localizes at the cell poles and also with maturing PG. (B) TADA initially labels cell poles and cell division septum. Scale bar is 5 µm.

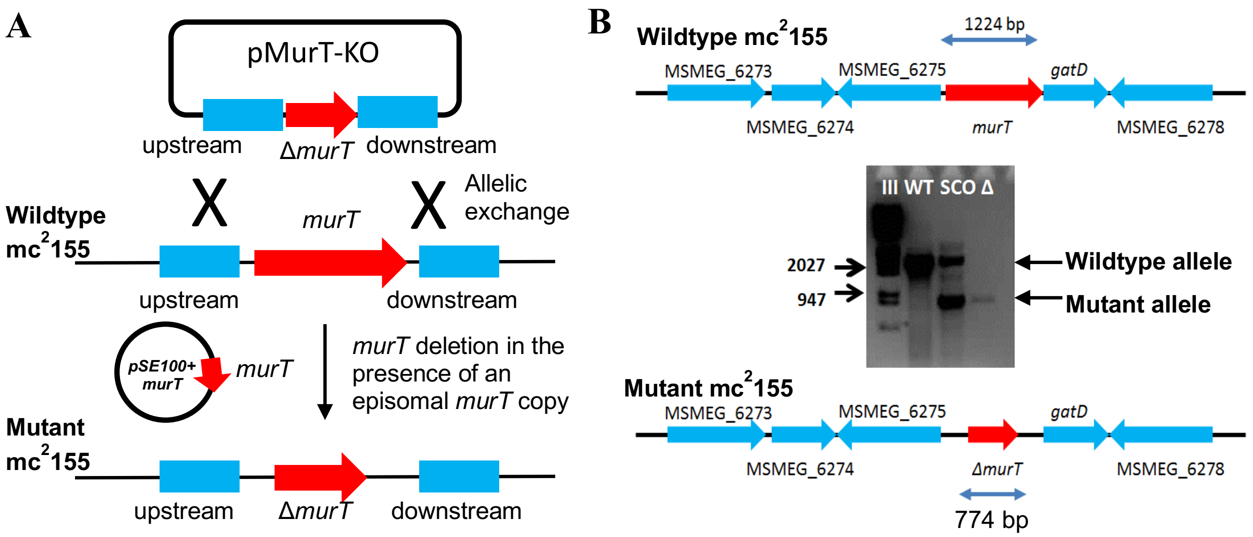

Supplementary Figure S2. Knockout of *murT* from a *M. smegmatis* strain carrying a second episomal copy of *murT*. (A) Schematic diagram showing the *murT* knockout strategy in *M. smegmatis* mc^{2}155. (B) The genome map of the wildtype and mutant *murT* alleles with an agarose gel depicting the PCR products of the wildtype (WT) and mutant (Δ) *murT* alleles in the wildtype strain, the single cross-over (SCO) strain and the mutant (Δ) strain. III – DNA marker III.

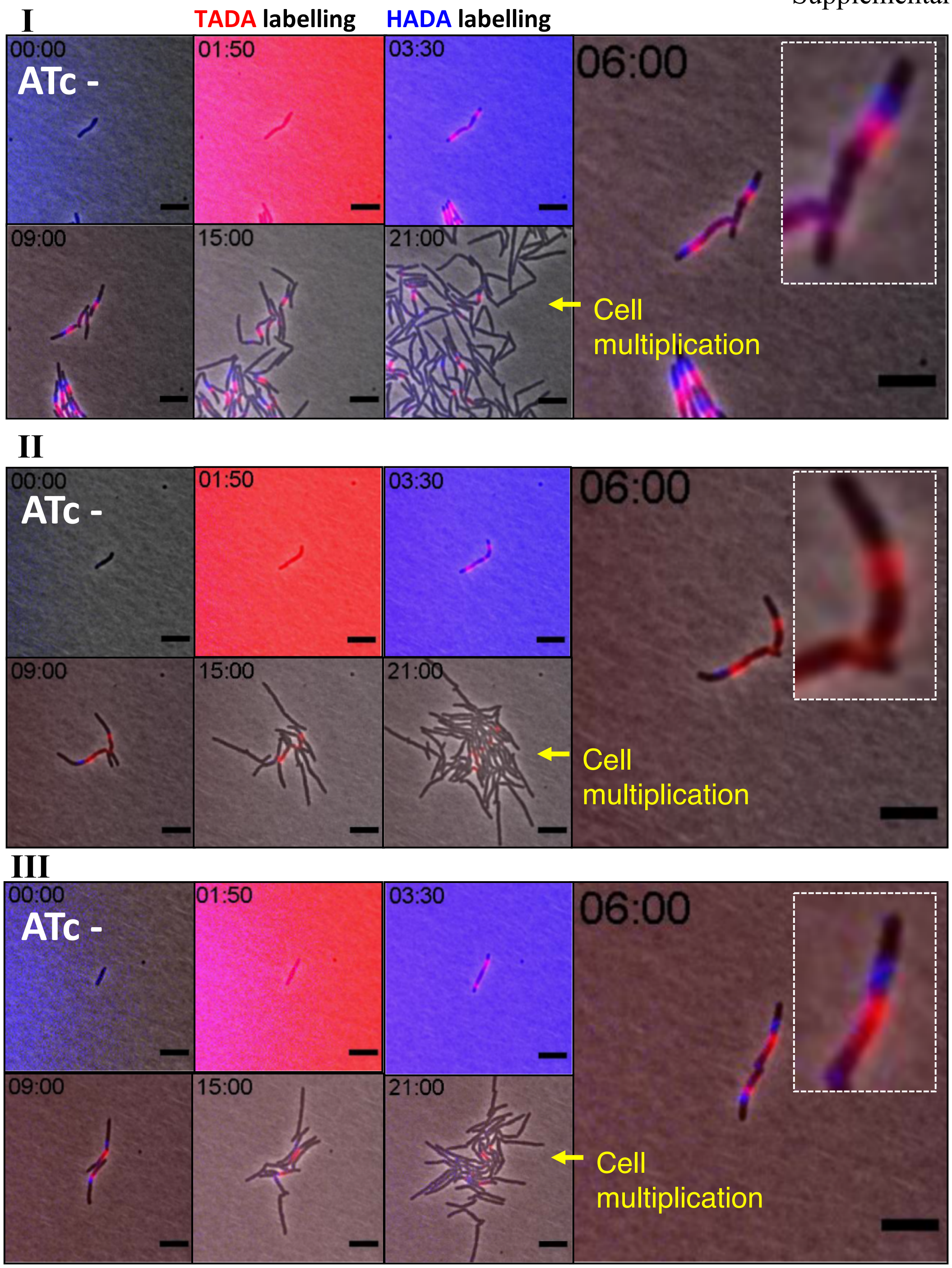

Supplementary Figure S3. Micrographs of mc^{2}155::CRISPRi-MurT-GatD without CRISPRi induction and growth analysis of mc^{2}155::CRISPRi-MurT-GatD with/without CRISPRi induction. (A) Time-lapse micrographs of the MurT-GatD depletion strain without CRISPRi activation (ATc-) pulse-chase labelled with FDAAs (TAMRA-D-Alanine [TADA] and HCC-D-Alanine [HADA]). The cells were grown first in no-label media only and subsequently labelled for 15 min with media supplemented with 1 mM TADA. The TADA supplemented media was washed out with no-label media and the cells were later labelled for 15 min with 1 mM HADA supplemented media which was later replaced with no-label media. Time-lapse microscopy of the TADA & HADA pulse-chase labelled cells was performed for 24 hours. Scale bar is 5 µm.

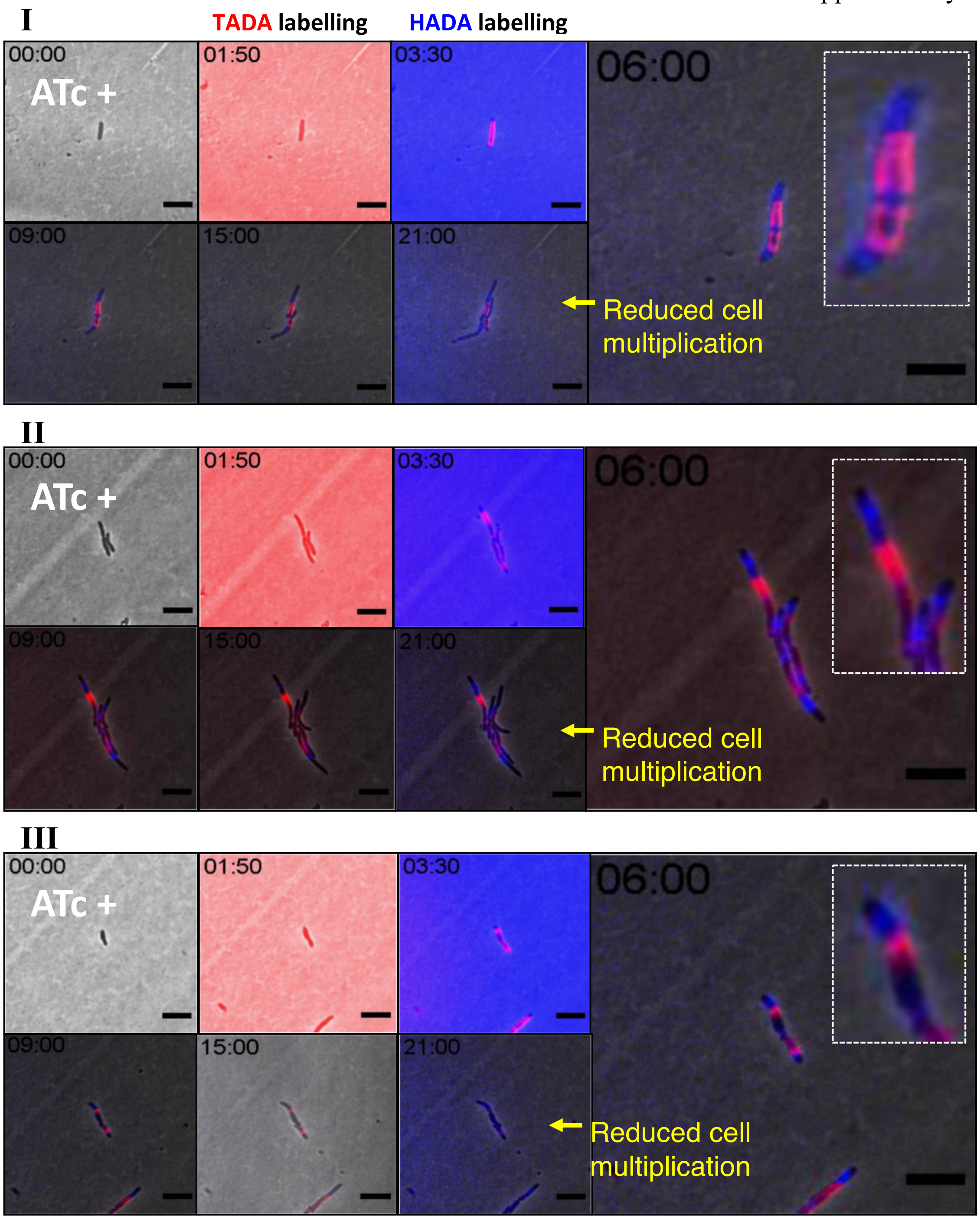

Supplementary Figure S4. Time-lapse micrographs of the MurT-GatD depletion strain with CRISPRi activation (ATc+) and pulse-chase labelling with FDAAs (TAMRA-D-Alanine [TADA] and HCC-D-Alanine [HADA]). The cells were grown first in no-label media (supplemented with 200 ng/ml ATc) and subsequently labelled for 15 min with media supplemented with 1 mM TADA and 200 ng/ml ATc. The TADA supplemented media was washed out with no-label media and the cells were later labelled for 15 min with media supplemented with 1 mM HADA and 200 ng/ml ATc which was later replaced with no-label media supplemented with 200 ng/ml ATc. Time-lapse microscopy of the TADA & HADA pulse-chase labelled MurT-GatD depletion cells was performed for 24 hours. CRISPRi mediated depletion of MurT-GatD resulted in reduced cell growth. Scale bar is 5 μm.

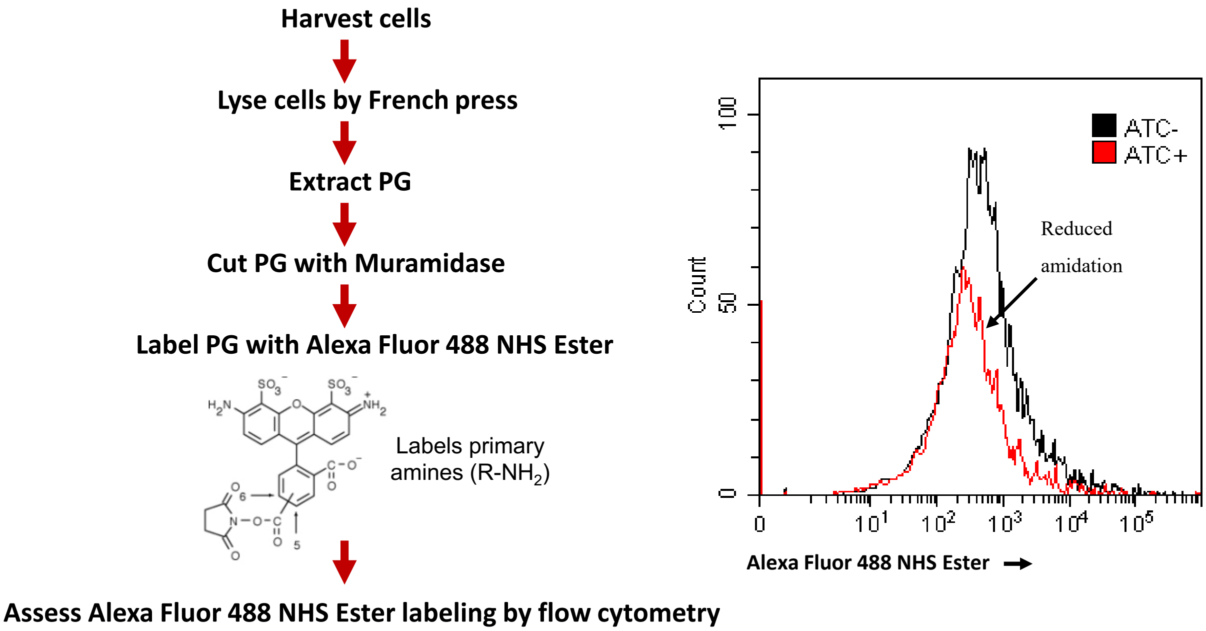

Supplementary Figure S5. **Depletion of MurT and GatD causes reduced PG amidation**. (A) Flow chart representation of the protocol used for assessing PG amidation in MurT-GatD depleted cells by Alexa Fluor 488 NHS Ester labelling of PG in comparison with control cells (ATc-). The Alexa Fluor 488 NHS Ester labels primary amines (R-NH_2) also found in PG as a result of amidation. (B). Flow cytometry quantification of Alexa Fluor 488 NHS Ester labeled PG from MurT-GatD depleted cells in comparison to the no ATc control cells. MurT-GatD depletion causes decreased PG amidation which results in decreased labeling with Alexa Fluor 488 NHS Ester.

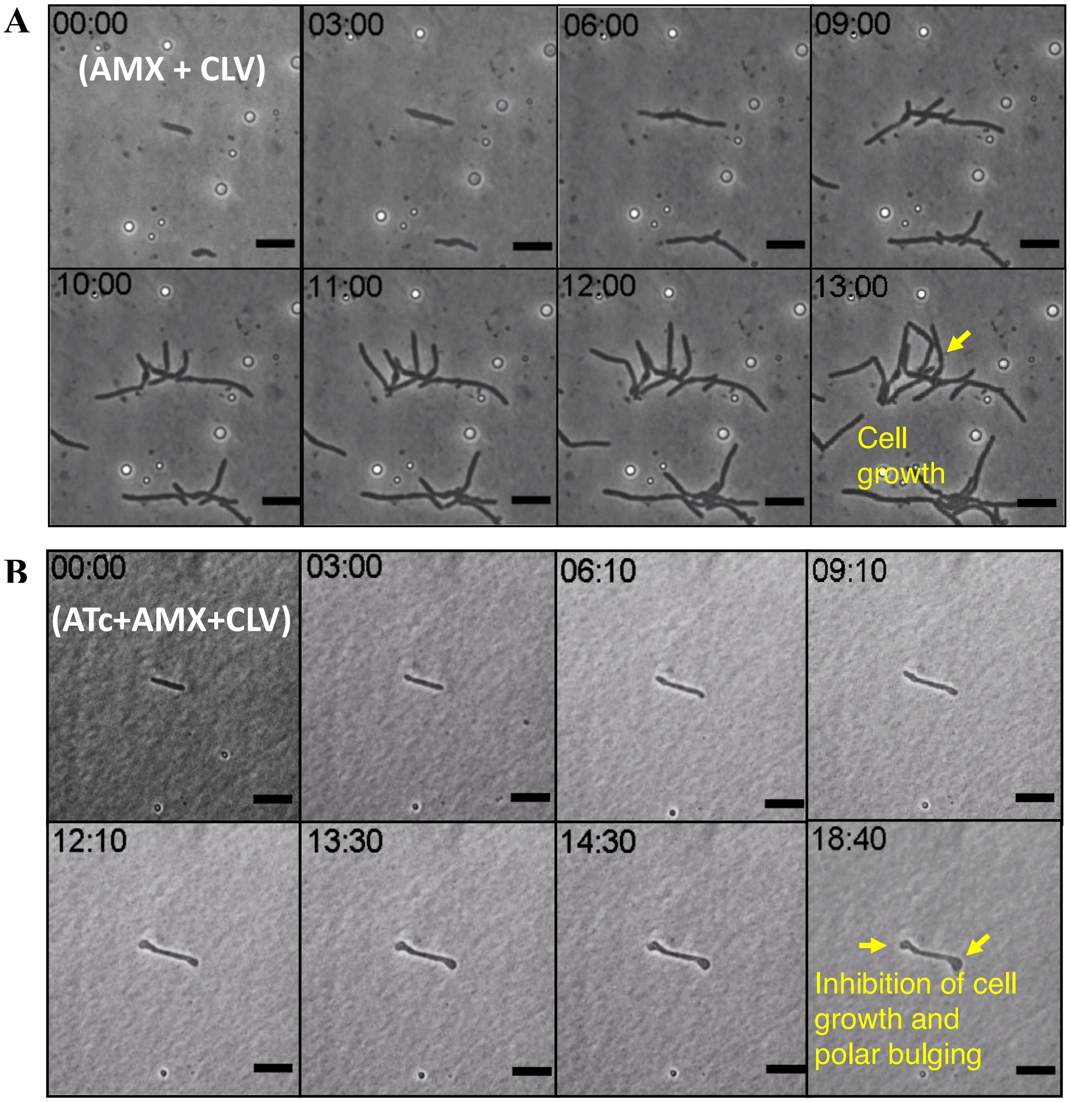

Supplementary Figure S6. Time-lapse microscopic analysis of amoxicilin (AMX) and clavulanate (CLV) treated MurT-GatD depletion strain cells with/without CRISPRi activation. (A) Micrographs of amoxicilin (AMX) and clavulanate (CLV) treated MurT-GatD depletion strain cells with/without CRISPRi activation. The cells were grown in media supplemented with sublethal concentrations of amoxicillin (20 µg/ml) and clavulanate (10 µg/ml). Growth of the cells in sublethal concentrations of amoxicillin and clavulanate did not cause any morphological changes. (B) Micrographs of amoxicillin and clavulanate treated MurT-GatD depletion strain cells with CRISPRi activation (ATc+). The cells were grown in media supplemented with 200 ng/ml ATc and amoxicillin (20 µg/ml) and clavulanate (10 µg/ml). Growth of the MurT-GatD depletion cells in sublethal concentrations of amoxicillin and clavulanate caused cell pole bulging and cell lysis. Time-lapse microscopy of the MurT-GatD depletion cells was performed for 24 hours. Scale bar is 5 µm.
