# Supplementary material for: Amidation of glutamate residues in mycobacterial peptidoglycan is essential for cell wall cross-linking
Source: Front Cell Infect Microbiol. 2023 Aug 24;13:1205829. doi: 10.3389/fcimb.2023.1205829 (PMC10484409; doi:10.3389/fcimb.2023.1205829)
Supplement: Supplementary file 14 [file Table_1.docx]

**Tables**

**Table A1.** Bacterial strains created and/or used in this study.

| **Strain** | **Description** | **Source/**  **Reference** | |  |
| --- | --- | --- | --- | --- |
| *Escherichia coli* DH5α | *SupE44 ΔlacU169 hsdR17 recA1 endA1 gyrA96 thi-1 relA1* | Promega | |  |
| mc^2^155 | High frequency transformation mutant of *M. smegmatis* ATCC 607 | (Snapper et al., 1990) |  |  |
| mc^2^155::pse100+MurT | Derivative of mc^2^155 carrying the pse100+MurT plasmid. Hyg^R^ | This study |  |  |
| ΔMurT::pse100+MurT | Derivative of mc^2^155::pse100+MurT with an unmarked deletion in the native MSMEG_6276 (*murT*) gene. Hyg^R^ | This study |  |  |
| mc^2^155::CRISPRi-MurT-GatD | A CRISPRi modified derivative of mc^2^155 carrying plasmid PLRJ962+MSMEG_6276sgRNA integrated at the *attB* site and expressing a sgRNA targeting the 5’ region of *murT* (MSMEG_6276). Kan^R^ | This study | | |
| mc^2^155::MurT-rseGFP | Derivative of mc^2^155 expressing a rseGFP tagged wildtype allele of MSMEG_6276 (*murT*) at the *tRNA* glycine site. Constructed by electroporation of plasmid pMV306H+rseGFP into mc^2^155. Hyg^R^ | This study | | |
| mc^2^155::MurT-rseGFP-mRFP-GatD | Derivative of mc^2^155::MurT-rseGFP expressing a mRFP tagged wildtype allele of MSMEG_6276 *(gatD*) at the *attB* site. Constructed by electroporation of plasmid pTweety+mRFP-GatD into mc^2^155::MurT-rseGFP. Hyg^R^ , Kan^R^ | This study | | |
| mc^2^155::CRISPRi-MurT-GatD+PknB-FLAG | Derivative of mc^2^155::CRISPRi-MurT-GatD expressing a FLAG tagged wildtype allele of MSMEG_0028 (*pknB*) at the *tRNA* glycine site. Constructed by electroporation of plasmid pFLAGEM +PknB into mc^2^155::CRISPRi-MurT-GatD. Kan^R^,Hyg^R^ | This study | | |
| mc^2^155:: mRFP-PknB | Derivative of mc^2^155 expressing a mRFP tagged wildtype allele of MSMEG_0028 (*pknB*) at the *tRNA* glycine site. Constructed by electroporation of plasmid pMV306H +mRFP-PknB into mc^2^155. Hyg^R^ | This study | | |
| mc^2^155::CRISPRi-MurT-GatD+mRFP-PknB | Derivative of mc^2^155::CRISPRi-MurT-GatD expressing a mRFP tagged wildtype allele of MSMEG_0028 (*pknB*) integrated at the *tRNA* glycine site. Constructed by electroporation of plasmid pMV306H +mRFP-PknB into mc^2^155::CRISPRi-MurT-GatD. Kan^R^,Hyg^R^ | This study | | |
| Mtb H37RvS | Virulent laboratory isolate ATCC 25618 | Laboratory Stock | | |

Hyg^R^: Hygromycin resistance, Kan^R^: Kanamycin resistance

**Table A2.** Plasmids created and/or used in this study.

| **Plasmids** | **Description** | **Source/**  **Reference** |
| --- | --- | --- |
| pse100 | *E. coli*-mycobacterial episomal shuttle vector containing origins of *E. coli* (pMB1) and mycobacteria (pAL500) and a multiple cloning site flanked by two transcriptional terminators. Hyg^R^ | (Guo et al., 2007) |
| pse100+MurT | Derivative of pse100 carrying a wildtype *murT* allele | This study |
| p2NIL | *E. coli* cloning vector. Kan^R^ | (Gordhan and Parish, 2001) |
| pMurT-KO | Derivative of p2NIL carrying the delta-*murT* deletion allele and the lacZ and *sacB* genes. Kan^R^ | This study |
| PLRJ962 | CRISPR interference vector for programmable transcriptional repression in mycobacteria using an orthogonal CRISPR interference platform. This plasmid expresses an ATc inducible *dCas9* from *Streptococcus thermophiles*. Kan^R^ | (Rock et al., 2017) |
| PLRJ962+MSMEG_6276sgRNA | A derivative of PLRJ962 expressing an ATc inducible *dCas9* from *S. thermophiles* and a (MSMEG_6276 [*murT*]) targeting sgRNA. Kan^R^ | This study |
| pMV306H | Genetic complementation vector, which integrates at the *attB* site in the mycobacterial genome. Hyg^R^ | CBTBR |
| pMV306H+rseGFP | Derivative of pMV306H carrying the rse-*gfp* allele. Hyg^R^ | Melissa Chengalroyen (CBTBR) |
| pMV306H+MurT-rseGFP | Derivative of pMV306H+rseGFP carrying the wildtype MSMEG_6276 (*murT*) allele linked at the C-terminus with rseGFP. Hyg^R^ | This study |
| pTweety | Complementation vector carrying an integrase gene. Kan^R^ | (Stover et al., 1991) |
| pTweety-mRFP | Derivative of pTweety carrying the *mRFP* allele. Kan^R^ | Melissa Chengalroyen (CBTBR) |
| pTweety-mRFP+GatD | Derivative of pTweety+mRFP carrying the MSMEG_6277 (*gatD*) allele fused to mRFP at the N-terninus. Kan^R^ | This study |
| pFLAGEM | *E. coli*- mycobacterial episomal shuttle vector carrying the 3X FLAG epitope sequence and the Tet-operator. Hyg^R^ | (Narrandes et al., 2015) |
| pFLAGEM+PknB | Derivative of pFLAGEM carrying a FLAG tagged wildtype allele of MSMEG_0028 (*pknB*) | This study |
| pMV306H-mRFP-PknB | Derivative of pMV306H+mRFP carrying the MSMEG_0028 (*pknB*) allele fused to mRFP at the N-terninus. Kan^R^ |  |

Hyg^R^: Hygromycin resistance, Kan^R^: Kanamycin resistance

**Table A3.** CRISPRi generated strain details

| CRISPRi Strain | Gene Name | PAM Sequence | sgRNA Targeting Sequence and primers for cloning (5’-3’) |
| --- | --- | --- | --- |
| 1. mc^2^155::CRISPRi-MurT-GatD | MSMEG_6276-MSMEG_6277  Operon | TGAGCAG | GAGCTGATCACGCGACAGAT  Primers  Fwd: GAGCTGATCACGCGACAGAT  Rev: ATCTGTCGCGTGATCAGCTC |
